# Association of Lipid Levels With the Prevalence of Hypertension in Chinese Women: A Cross-Sectional Study Based on 32 Health Check Centers

**DOI:** 10.3389/fendo.2022.904237

**Published:** 2022-07-07

**Authors:** Guizhi Deng, Yunjie Li, Wenke Cheng

**Affiliations:** ^1^ Department of Cardiovascular Medicine, The Third Hospital of Nanchang, Nanchang, China; ^2^ Department of Gastrointestinal Endoscopy, The Third Hospital of Nanchang, Nanchang, China; ^3^ Medical Faculty, University of Leipzig, Leipzig, Germany

**Keywords:** hypertension, total cholesterol, low-density cholesterol, triglycerides, high-density lipoprotein cholesterol, lipids

## Abstract

**Background:**

Dyslipidemia is strongly associated with the development of hypertension. In our previous study, it was shown that elevated TC, LDL-c, and non-HDL-c were associated with the prevalence of hypertension in Chinese men, whereas the relationship between HDL-c and hypertension shifted from no association to a positive association after adjusting for the BMI. To further accumulate epidemiological evidence in Asian women, this study aimed to investigate the relationship between lipid profile and prevalence of hypertension in Chinese adult women.

**Methods:**

This is a cross-sectional study including 54,099 Chinese women aged>20 years at 32 health screening centers in 11 cities from 2010-2016. The original data were obtained from DATADRYAD database (www.datadryad.org). Besides, the overall women were classified into non-hypertensive and hypertensive groups based on baseline blood pressure levels. Differences between the two groups were examined by Man-Whitney test or Chi-square test. Spearman’s correlation coefficient was employed to evaluate the correlation between systolic blood pressure (SBP), diastolic blood pressure (DBP) and lipid profiles. Multivariate logistic regression was performed to estimate the relationship between different lipid levels and the prevalence of hypertension. Odds ratios (ORs) and 95% confidence intervals (CIs) indicated the risk of lipid and hypertension. Bayesian model (BN) model was constructed to further assess the relationship between baseline characteristics and the prevalence of hypertension, as well as the importance of each variable for the prevalence of hypertension.

**Results:**

Compared to the non-hypertensive population, the hypertensive population was older, and had the higher body mass index (BMI), total cholesterol (TC), low-density lipoprotein cholesterol (LDL-c), serum creatinine (Scr), fasting blood glucose (FPG), blood urea nitrogen (BUN), alanine aminotransferase (ALT), aspartate aminotransferase (AST), and non-high-density lipoprotein cholesterol (non-HDL-c), but HDL-c and the presence concerning the family history of diabetes were lower. Multivariate logistic regression analysis revealed that TC, LDL-c, and non-HDL-c showed a positive trend with hypertension risk (*p* for trend < 0.05) whereas TC and HDL-c were not significantly associated with hypertension prevalence. Moreover, each 1 mg/dl increase in TC, LDL, and non-HDL hypertension prevalence increased by 0.2% [1.002 (1.000-1.003)], 0.2% [1.002 (1.000-1.004)], and 0.2% [1.002(1.001-1.004)], respectively. BN suggested that the importance of age, BMI, FPG, non-HDL-c on the prevalence of hypertension was 52.73%, 24.98%, 11.22%, and 2.34%, respectively.

**Conclusion:**

Overall, in Chinese adult women, TC, LDL-c and non-HDL-c levels were higher and HDL-c level was lower in the hypertensive population, whereas TG did not differ significantly from the non-hypertensive population. Meanwhile, TC, LDL-c, and non-HDL-c were positively associated with prevalence of hypertension, and HDL-c was negatively associated with prevalence of hypertension but became nonsignificant after full adjustment for variables. Moreover, BN model suggested that age, BMI, FPG, and non-HDL-c had a greater effect on the development of hypertension.

## Introduction

Arterial hypertension is not only a critical challenge to global public health today, but also a major risk factor for cardiovascular disease (CVD) (1, 2). Currently, hypertension is one of the most important causes of death in the world and the third leading cause of disability (1). The prevalence of hypertension is increasing year by year, the number of people suffering from hypertension is expected to reach 1.56 billion in 2025, and one-third of the adults in China suffer from hypertension (3, 4). In clinical practice, elevated blood pressure is commonly observed to coexist with dyslipidemia, independent of gender and age, constituting an additional risk factor for CVD (5). Therefore, early identification of risk factors for hypertension and effective prevention are essential to lower the public health burden.

A growing number of epidemiological studies have shown that dyslipidemia is strongly associated with the development of hypertension (6–11), but most of the studies are from Europe or the United States, with fewer studies from Asia (12, 13). Although the results of these studies consistently show a strong association between dyslipidemia and the prevalence of hypertension, there still exist differences in specific lipid profile patterns. Hence, the relationship between lipid profile patterns and hypertension may vary across different ethnics as well as subgroup populations with clinical characteristics. Our previous study revealed that elevated TC, LDL-c, and non-HDL-c were associated with the prevalence of hypertension in Chinese men, whereas the relations between HDL-c and hypertension shifted from no association to a positive association after adjusting for the BMI. In addition, BN models showed that age, BMI, FPG and TC were strongly related to the prevalence of hypertension (14). Therefore, to further accumulate epidemiological evidence in Asian populations, the aim of this study was to investigate the relationship between lipid profile and prevalence of hypertension in Chinese adult women.

## Methods

### Study Design and Data Extraction

The design of present study is a cross-sectional study. The original data were downloaded freely from the DATADRYAD database (www.datadryad.org), provided by Chen et al. (15), and we performed a secondary analysis. Specifically, totally 211,833 Chinese adults were recruited at 32 health check centers in 11 Chinese cities (Shanghai, Beijing, Nanjing, Suzhou, Shenzhen, Changzhou, Chengdu, Guangzhou, Hefei, Nantong, and Wuhan) from 2010 to 2016. The study by Chen et al. aimed to assess the association of body mass index (BMI) and age with the prevalence of diabetes in Chinese adults. All participants completed a detailed questionnaire at their first visit to the health check center, including demographics, lifestyle, and family history of chronic disease. In addition, participants’ clinical information such as weight, height, and blood pressure were measured by trained staff. The following parameters, namely, total cholesterol (TC), high-density lipoprotein cholesterol (HDL-c), low-density lipoprotein cholesterol (LDL-c), serum creatinine (Scr), fasting blood glucose (FPG), blood urea nitrogen (BUN), glutamate transaminase (ALT), and aspartate aminotransferase (AST) can be obtained directly by a uniform autoanalyzer (Beckman 5800). Besides, BMI was calculated by dividing the weight by the square of the height. All data were collected under standardized conditions following a uniform procedure.

Under the CC0 1.0 Universal (CC0 1.0) Public Domain Exclusivity License, these data could be used for secondary analysis without infringing on the authors’ rights because Chen et al. have waived all copyright and related ownership of the original data. Apart from that, the original study was approved by the Rich Healthcare Group Review Board, the baseline information was retrieved retrospectively, and the Rich Healthcare Group Review Board waived the requirement for informed consent (16). Moreover, this study complied with the Declaration of Helsinki.

### Study Population

**Figure 1:**
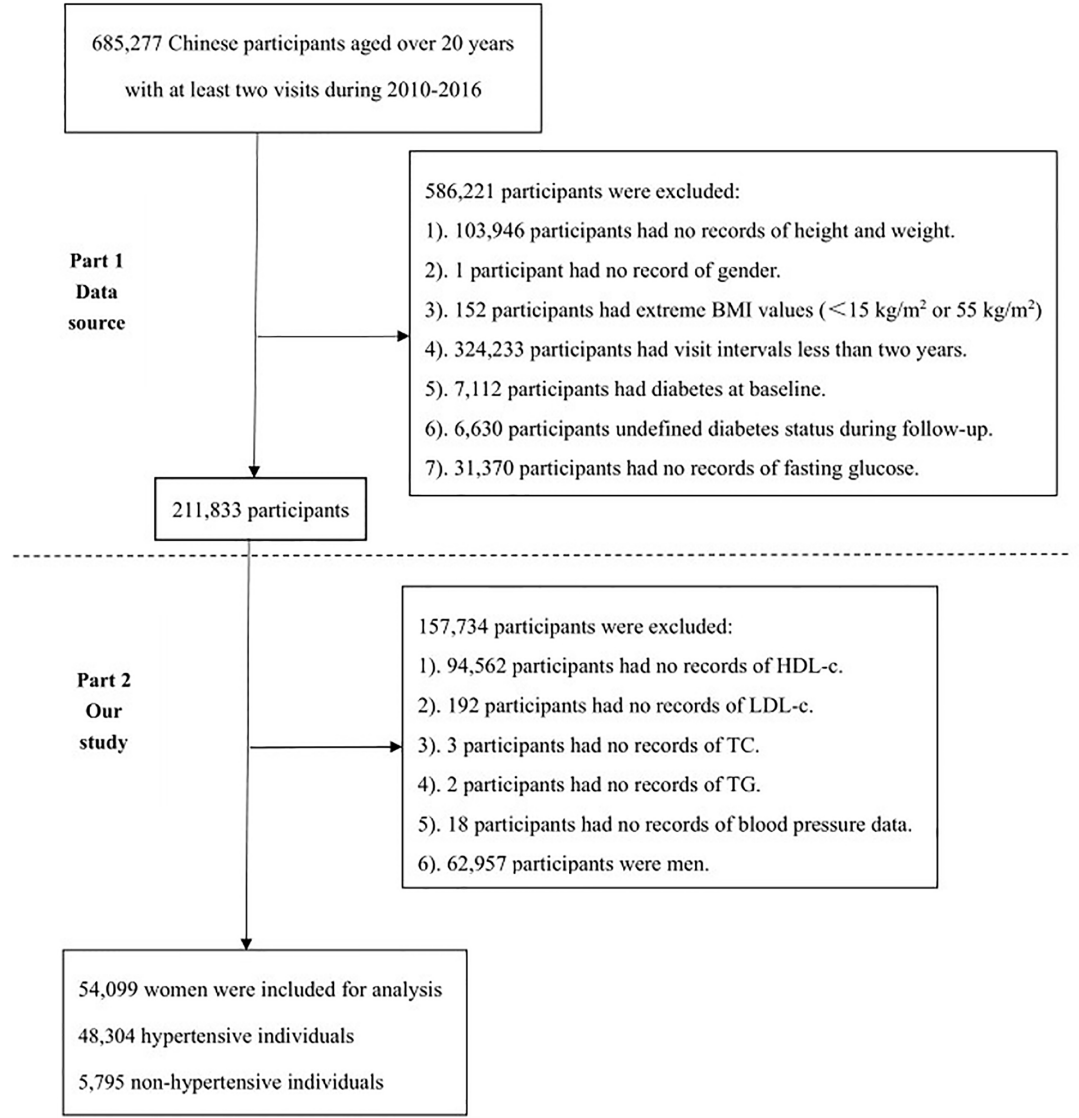
The flowchart of study participants.

As shown in **Figure 1**, the flowchart of study population selection consisted of two parts. The first part is the selection process of the Chen et al. containing a total of 211,833 participants. The second part shows the selection process of the current study. Only lipid profiles with complete information on TC, TG, LDL, and HDL-c were involved in our analysis. Based on the first part, in which 157.734 participants were further excluded for the following specific reasons. 1). 94,562 participants had no records of HDL-c. 2). 192 participants had no records of LDL-c. 3). 3 participants had no records of TC. 4). 2 participants had no records of TG. 5). 18 participants had no records of blood pressure data. 6). 62,957 participants were men. Ultimately, 54,099 Chinese adult women including 48,304 hypertensive and 5,795 non-hypertensive individuals were involved in the current study for analysis.

### Exposure of Interest and Outcomes

The exposure of interests in this study included lipid profiles and blood pressure levels. Lipid profiles included TC, TG, LDL-c, HDL-c, and non-HDL-c. non-HDL-c was obtained by subtracting HDL from plasma TC levels. Blood pressure was obtained from office blood pressure measurements by trained staff using a standard mercury sphygmomanometer. Referring to the Chinese Guidelines for the Prevention and Treatment of Hypertension (2018 version) (17), participants fell into hypertensive and non-hypertensive groups according to one office blood pressure measurement (baseline blood pressure levels). Hypertensive group was defined as individuals with systolic blood pressure (SBP)≥140 mmHg or diastolic blood pressure (DBP)≥90 mmHg, while non-Hypertensive group was defined as individuals with SBP<140 mmHg and DBP<90 mmHg. In the meanwhile, hypertension was further divided into three groups, Grade I (SBP 140-159 or DBP 90-99 mmHg), Grade II (SBP 160-179 or DBP 99-109 mmHg) and Grade III (SBP≥180 or DBP≥110 mmHg). The first outcome of this study was to assess the differences in lipid profiles between hypertensive and non-hypertensive populations. The second outcome was to further assess the relationship between potential baseline characteristics including lipid profiles and the prevalence of hypertension by constructing a Bayesian network (BN) model.

### Statistical Analyses

Due to the skewed distribution of the baseline characteristic data, all continuous variables are expressed as median and interquartile range (IQR). Dichotomous variables are expressed as number (proportions). Differences between the two groups of continuous variables were examined by Man-Whitney test, and differences between the two groups of dichotomous variables were examined by Chi-square test. Moreover, differences in lipids profiles between different blood pressure groups (more than three groups) were analyzed using Kruskal-Wallis one-way analysis of variance (ANOVA) and Dunn’s test. Spearman’s correlation coefficient was applied to evaluate the correlation between SBP, DBP and lipid profiles. Subgroup analysis was performed by age (<60 and≥60 years), BMI (<23 and≥23 kg/m^2^) and ALT (<40 and≥40U/L). Specifically, age≥60 years was defined as “older” and <60 years as “young-middle-aged” (18); BMI≥23kg/m^2^ was defined as “overweight/obese” and <23kg/m^2^ as “normal/underweight” according to Asian-specific criteria (19); ALT≥40U/L was defined as “elevated” and <40U/L as “normal” (20). In addition, lipid profiles were grouped into quartiles as continuous variables. Multivariate logistic regression was performed to estimate the relationship between different lipid levels and the prevalence of hypertension, while trend for *p* was obtained. Statistical analyses were performed using SPSS 26.0 and GraphPad 9.0, and two-tailed *p*<0.05 was considered statistically significant.

The BN model, which usually combines probability theory and graph theory, is one of the probabilistic graphical models that reveal the probabilistic dependence between variables (nodes). The BN model was constructed to further assess the relationship between baseline characteristics and the prevalence of hypertension, as well as the importance of each variable for the prevalence of hypertension. The model was built based on the tree augmented native (TAN) algorithm in the modeling section of SPSS Modeler (version 18.0), and the parameter learning method was chosen as Bayesian adjustment of small cell counts (21). Arrows connecting two nodes indicate that the two random variables are causally or unconditionally independent. If two nodes do not have arrows, the random variables are conditionally independent (22). Based on these advantages of the BN model described above, the interaction of baseline characteristics and lipid profiles of participants with the prevalence of hypertension, and the significance of these aspects for the prevalence of hypertension were further investigated.

## Results

The median age of the overall women was 41 (34-52) years old, with the hypertensive population aged 59 (50-66) years old and the non-hypertensive population aged 39 (33-50) years old, and the age of the hypertensive population was older than the non-hypertensive population (p<0.001). In addition, BMI, FPG, ALT, AST, BUN, Scr, TC, LDL-c and non-HDL-c of hypertensive individuals were higher than those of non-hypertensive individuals, as shown in **Table 1**. However, family history of diabetes presence and HDL were lower in hypertensive individuals, while there existed no significant differences in presence of current smoker and drinker, and TG between the two groups.

**Table 1:** Baseline information of the adult women*.

	Total (n = 54,099)	Non-hypertension (n = 48,304)	Hypertension (n = 5,795)	P value
Age, years	41 (34-52)	39 (33-50)	59 (50-66)	<0.001
Family history of diabetes (%)	1610 (3)	1488 (0.03)	122 (0.02)	<0.001
Current smoker (%)	17 (0.03%)	17 (0.03%)	0 (0%)	0.153
Current drinker (%)	17 (0.03%)	16 (0.03%)	1 (0.017%)	0.520
SBP (mmHg)	112 (103-125)	110 (102-120)	146 (140-155)	<0.001
DBP (mmHg)	70 (64-78)	69 (63-75)	88 (81-94)	<0.001
BMI (kg/m2)	21.8 (20-24)	21.6 (19.9-23.7)	24.2 (22.1-26.4)	<0.001
FPG (mmol/L)	4.88 (4.51-5.22)	4.9 (4.56-5.23)	5.18 (4.8-5.6)	<0.001
ALT (U/L)	14 (11-19)	13.8 (10.9-18.2)	17 (13-24)	<0.001
AST (U/L)	20 (17.2-24.0)	20 (17-23.7)	23 (19.6-27.6)	<0.001
BUN (mmol/L)	4.24 (3.58-5.04)	4.21 (3.55-4.99)	4.59 (3.88-5.42)	<0.001
Scr (μmol/L)	57.3 (51.9-63.5)	58 (52.4-64.5)	61 (54-69)	<0.001
TC (mg/dl)	180.54 (158.89-204.9)	177.84 (158.51-202.19)	197.17 (173.97-224.23)	<0.001
TG (mg/dl)	97.46 (68.22-147.08)	97.46 (67.34-145.30)	98.34 (68.22-147.08)	0.319
LDL-c (mg/dl)	102.84 (86.99-121.01)	101.68 (85.6-119.07)	114.82 (97.81-132.99)	<0.001
HDL-c (mg/dl)	56.06 (48.71-63.79)	55.28 (48.71-63.02)	55.28 (48.33-62.24)	<0.001
Non-HDL-c (mg/dl)	122.94 (103.61-146.52)	121.39 (103.22-143.43)	141.3 (120.62-165.85)	<0.001

*Continuous data are expressed as median (interquartile range) due to the skewed distribution.

The p-value is a comparison between the normotension and hypertension groups.

FPG, fasting plasma glucose; TG, triglycerides; TC, total cholesterol; HDL-c, high-density lipoprotein cholesterol; LDL, low-density lipoprotein cholesterol; Scr, serum creatinine; BUN, blood urea nitrogen; ALT, alanine aminotransferase; AST, aspartate aminotransferase; BMI, Body mass index.

### Correlation Between SBP, DBP and Lipid Profiles

Spearman correlation analysis showed that SBP was positively correlated with TC(r=0.27, p<0.001), LDL-c(r=0.21, p<0.001), and non-HDL-c(r=0.26, p<0.001), negatively correlated with HDL(r=-0.06, p<0.001), and not significantly correlated with TG(p=0.42). Similarly, DBP was positively correlated with TC(r=0.19, p<0.001), LDL-c(r=0.18, p<0.001), and non-HDL-c(r=0.22, p<0.001), negatively correlated with HDL(r=-0.06, p<0.001), and not significantly correlated with TG(p=0.056).

### Subgroup Analysis of Differences in Lipid Profiles Between the Hypertensive and Non-Hypertensive Groups

In the population aged<60 years, the hypertensive population had higher levels of TC, LDL-c, and non-HDL-c and lower levels of HDL-c, with no significant differences in TG **Table 2**. Similarly, in those aged≥60 years, HDL-c was lower and non-HDL was higher in the hypertensive population, whereas LDL, TC, and TG were not significantly different. In those with BMI <23 or ≥23 kg/m^2^, TC, LDL-c, and non-HDL-c levels were higher in the hypertensive group, whereas HDL-c and TG were not significantly different from those in the non-hypertensive group. Similarly, in those with ALT <40 or ≥40 U/L, elevated TC, LDL-c, and non-HDL-c were observed in the hypertensive population with no significant difference in TG, yet lower HDL-c levels.

**Table 2:** Subgroup analysis of lipid profiles differences between non-hypertension and hypertension according to age, BMI, and ALT*.

	Non-hypertension	Hypertension	P-value	Non-hypertension	Hypertension	P-value
**Age**	<60 years			≥60 years		
TC (mg/dl)	175.52 (155.8-197.94)	190.59 (168.17-215.72)	<0.001	208.76 (185.57-233.89)	210.31 (187.11-235.83)	0.054
TG (mg/dl)	97.46 (68.22-146.19)	97.9 (67.34-144.2)	0.921	97.46 (68.22-145.30)	100.12 (67.34-149.73)	0.205
LDL (mg/dl)	99.36 (85.05-116.37)	110.37 (93.94-128.35)	<0.001	122.17 (104.77-141.50)	122.17 (104.77-141.5)	0.897
HDL-c (mg/dl)	56.06 (48.71-64.18)	53.74 (46.78-61.86)	<0.001	56.83 (49.1-64.56)	55.67 (47.55-63.4)	<0.001
Non-HDL-c (mg/dl)	118.3 (110.52-139.18)	135.31 (114.82-159.28)	<0.001	151.55 (129.12-174.74)	153.48 (131.44-179.0)	<0.001
**BMI**	<23 kg/m^2^			≥23 kg/m^2^		
TC (mg/dl)	174.74 (155.03-197.94)	197.17 (173.58-224.23)	<0.001	185.57 (163.53-210.31)	201.03 (177.84-226.55)	<0.001
TG (mg/dl)	97.46 (67.34-147.08)	97.46 (66.45-145.97)	0.707	97.46 (68.22-145.30)	100.12 (68.22-147.08)	0.135
LDL (mg/dl)	98.97 (84.28)	114.05 (96.36-133.38)	<0.001	107.09 (90.85-126.03)	116.37 (99.36-135.31)	<0.001
HDL-c (mg/dl)	57.60 (50.26-65.72)	57.60 (49.97-65.72)	0.941	52.96 (46.39-60.7)	52.96 (46.01-61.08)	0.807
Non-HDL-c (mg/dl)	116.37 (98.97-137.63)	138.02 (115.98-163.92)	<0.001	131.06 (110.95-155.41)	147.29 (124.48-171.65)	<0.001
**ALT**	<40 U/L			≥40 U/L		
TC (mg/dl)	177.84 (157.73-201.3)	199.1 (175.13-225.0)	<0.001	192.91 (168.56-218.43)	209.54 (185.57-233.51)	<0.001
TG (mg/dl)	97.46 (68.22-147.08)	99.23 (67.34-147.96)	0.260	96.57 (66.45-143.53)	97.46 (67.34-139.10)	0.832
LDL (mg/dl)	100.9 (85.83-118.69)	115.59 (97.81-134.15)	<0.001	110.18 (93.17-130.09)	122.55 (104.77-140.34)	<0.001
HDL-c (mg/dl)	56.06 (49.1-64.18)	54.90 (47.55-62.63)	<0.001	53.35 (46.0-62.63)	52.19 (44.07-59.15)	<0.001
Non-HDL-c (mg/dl)	120.23 (101.68-142.66)	142.66 (120.62-168.17)	<0.001	137.24 (114.24-162.37)	156.57 (132.99-180.16)	<0.001

*Continuous data are expressed as median (interquartile range) due to the skewed distribution.

TG, triglycerides; TC, total cholesterol; HDL-c, high-density lipoprotein cholesterol; LDL, low-density lipoprotein cholesterol; ALT, alanine aminotransferase; BMI, Body mass index.

### Multivariate Logistic Regression Analysis of Lipid Profile and Prevalence of Hypertension

We grouped lipid profiles as continuous variables in quartiles, using the first quartile (Q1) as the reference, as displayed in **Table 3**. In the crude model, no variables were adjusted, and in model 1, only age was adjusted, whereas age and BMI were adjusted in model 2. In model 3, all variables including age, BMI, FPG, ALT, AST, Scr, BUN, smoking and drinking status, and family history of diabetes were fully adjusted. In all 4 models, there was no significant correlation between TG and prevalence of hypertension (p > 0.05). In addition, TC, LDL-c, and non-HDL-c showed a positive trend with hypertension risk (*p* for trend < 0.05). In model 3, each 1 mg/dl increase in TC, LDL, and non-HDL hypertension prevalence increased by 0.2% (1.002 [1.000-1.003]),0.2% (1.002 [1.000-1.004]), and 0.2% (1.002[1.001-1.004]), respectively. However, in the crude model, model 1 and model 2, HDL-c showed a negative trend with the prevalence of hypertension, whereas HDL-c was not significantly associated with the prevalence of hypertension after fully adjusting for all variables in model 4 (p=0.407).

**Table 3:** Multivariate logistic regression analysis of lipid profile levels and the risk of hypertension.

	Crude Model		Model 1		Model 2		Model 3	
TG	OR (95% CI)	*P*-value	OR (95% CI)	*P*-value	OR (95% CI)	*P*-value	OR (95% CI)	*P*-value
Q 1 (≤68.22 mg/dl)	Ref		Ref		Ref		Ref	
Q 2 (68.22-97.46 mg/dl)	0.94 (0.869-1.018)	0.127	0.984 (0.902-1.072)	0.707	0.975 (0.893-1.065)	0.577	1.023 (0.892-1.173)	0.745
Q 3 (97.46-146.19 mg/dl)	1.03 (0.956-1.112)	0.434	0.999 (0.919-1.085)	0.976	1.001 (0.920-1.09)	0.979	1.036 (0.907-1.183)	0.603
Q 4 (≥146.19 mg/dl)	0.999 (0.925-1.079)	0.975	0.978 (0.899-1.065)	0.612	0.987 (0.906-1.076)	0.770	1.038 (0.906-1.188)	0.593
Per 1 mg/dl increase	1.000 (1.000-1.001)	0.062	1.000 (1.000-1.000)	0.315	1.000 (1.000-1.001)	0.238	1.000 (1.000-1.001)	0.189
*p* for trend	0.487		0.712		0.926		0.569	
TC								
Q 1 (≤158.89 mg/dl)	Ref		Ref		Ref		Ref	
Q 2 (158.89-180.54 mg/dl)	1.562 (1.411-1.739)	<0.001	1.141 (1.024-1.27)	0.017	1.111 (0.996-1.240)	0.059	1.03 (0.87-1.211)	0.752
Q 3 (180.54-204.90 mg/dl)	2.556 (2.326-2.809)	<0.001	1.337 (1.207-1.48)	<0.001	1.269 (1.144-1.407)	<0.001	1.164 (0.997-1.36)	0.054
Q 4 (≥204.90 mg/dl)	4.548 (4.158-4.974)	<0.001	1.402 (1.269-1.55)	<0.001	1.322 (1.195-1.463)	<0.001	1.176 (1.009-1.371)	0.038
Per 1 mg/dl increase	1.015 (1.014-1.016)	<0.001	1.003 (1.002-1.004)	<0.001	1.003 (1.002-1.003)	<0.001	1.002 (1.000-1.003)	0.008
*p* for trend	<0.001		<0.001		<0.001		0.013	
LDL-c								
Q 1 (≤86.99 mg/dl)	Ref		Ref		Ref		Ref	
Q 2 (86.99-102.84 mg/dl)	1.55 (1.406-1.709)	<0.001	1.161 (1.046-1.289)	0.005	1.106 (0.994-1.230)	0.064	1.015 (0.862-1.195)	0.859
Q 3 (102.84-121.01 mg/dl)	2.413 (2.202-2.645)	<0.001	1.338 (1.212-1.478)	<0.001	1.247 (1.127-1.380)	<0.001	1.188 (1.018-1.387)	0.029
Q 4 (≥121.01 mg/dl)	3.969 (3.638-4.330)	<0.001	1.371 (1.245-1.511)	<0.001	1.265 (1.147-1.396)	<0.001	1.170 (1.005-1.362)	0.044
Per 1 mg/dl increase	1.017 (1.016-1.018)	<0.001	1.003 (1.002-1.004)	<0.001	1.003 (1.002-1.004)	<0.001	1.002 (1.000-1.004)	0.017
*p* for trend	<0.001		<0.001		<0.001		0.011	
HDL-c								
Q 1 (≤48.71 mg/dl)	Ref		Ref		Ref		Ref	
Q 2 (48.71-56.06 mg/dl)	0.808 (0.75-0.870)	<0.001	0.848 (0.782-0.920)	<0.001	0.927 (0.854-1.007)	0.074	0.993 (0.871-1.132)	0.919
Q 3 (56.06-63.79 mg/dl)	0.813 (0.754-0.877)	<0.001	0.797 (0.733-0.867)	<0.001	0.934 (0.857-1.017)	0.117	1.078 (0.944-1.231)	0.267
Q 4 (≥63.79 mg/dl)	0.694 (0.642-0.750)	<0.001	0.643 (0.590-0.701)	<0.001	0.871 (0.797-0.952)	0.002	0.894 (0.772-1.036)	0.137
Per 1 mg/dl increase	0.988 (0.986-0.991)	<0.001	0.986 (0.983-0.988)	<0.001	0.995 (0.993-0.998)	0.001	0.996 (0.991-1.000)	0.063
*p* for trend	<0.001		<0.001		0.004		0.407	
non-HDL-c								
Q 1 (≤103.61 mg/dl)	Ref		Ref		Ref		Ref	
Q 2 (103.61-122.94 mg/dl)	1.748 (1.573-1.943)	<0.001	1.224 (1.113-1.391)	<0.001	1.128 (1.007-1.264)	0.038	0.883 (0.742-1.051)	0.161
Q 3 (122.94-146.52 mg/dl)	2.934 (2.658-3.239)	<0.001	1.486 (1.337-1.653)	<0.001	1.293 (1.161-1.440)	<0.001	1.189 (1.011-1.398)	0.036
Q 4 (≥146.52 mg/dl)	5.624 (5.124-6.173)	<0.001	1.694 (1.528-1.878)	<0.001	1.382 (1.244-1.535)	<0.001	1.116 (0.950-1.311)	0.181
Per 1 mg/dl increase	1.017 (1.017-1.018)	<0.001	1.005 (1.004-1.006)	<0.001	1.003 (1.002-1.004)	<0.001	1.002 (1.001-1.004)	0.003
*p* for trend	<0.001		<0.001		<0.001		0.008	

OR, odds ratio; CI, confidence interval; Q, quartile; Model 1 adjust age; Model 2 adjust age and BMI. Model 3 adjust Model 2+ FPG (mmol/L), ALT, AST, BUN, Scr, smoking status (current smoker or not), drinking status (current drinker or not), family history of diabetes (Yes or No).

### Differences in Lipid Profiles at Different Blood Pressure Levels

**Figure 2:**
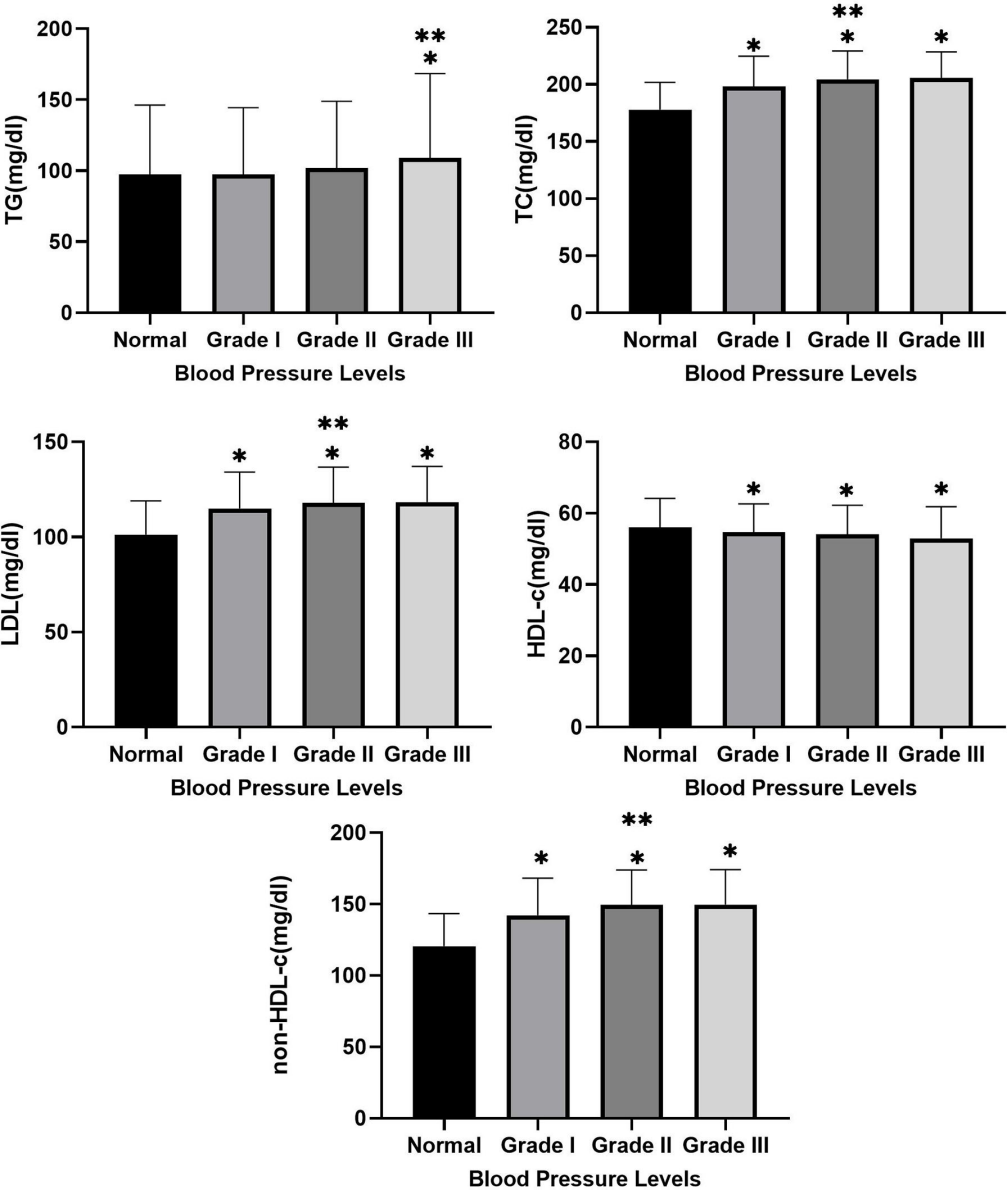
Between-group differences in TC, TG, LDL, HDL-c, and non-HDL at different blood pressure levels. * Indicates Grade I, Grade II and Grade III compared with normal group with p-value <0.05. ** Indicates Grade II and Grade III compared with Grade I with p-value <0.05.

As shown in **Figure 2**, TC levels were higher in the Grade III group than in the Normal and Grade I groups. Meanwhile, TC, LDL-c and non-HDL-c were higher in the Grade I, II and III groups than in the Normal group, and TC, LDL-c and non-HDL-c were higher in the Grade II group than in the Grade I group. In addition, the levels of HDL-c were higher in the Grade I, II and III groups than in the Normal group.

### Bayesian Network Model

**Figure 3:**
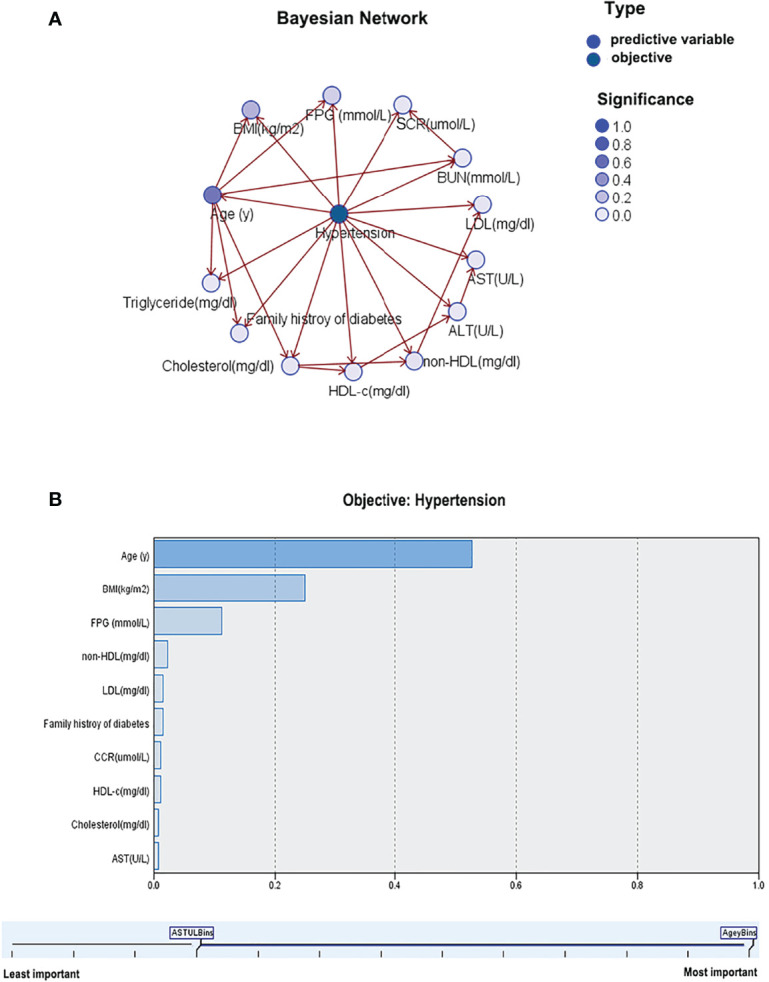
Bayesian network model for hypertension based on TAN algorithm **(A)**; Importance of predictors **(B)**.

As presented in **Figure 3A**, age indirectly affects hypertension prevalence and directly affects FPG, BMI, family history of diabetes, BUN, TG, and TC. In other words, FPG, BMI, family history of diabetes, BUN, TG, and TC indirectly affect hypertension prevalence through age as an intermediate variable. In the lipid profiles, TC indirectly affects the prevalence of hypertension, and TC directly influences HDL-c and non-HDL-c, while non-HDL-c directly affects LDL-c, thus TC and non-HDL-c indirectly affect hypertension prevalence as intermediate variables. The importance of age, BMI, FPG, non-HDL-c, LDL-c, family history of diabetes, Scr, HDL-c, TC, and AST on the prevalence of hypertension was 52.73%, 24.98%, 11.22%, 2.34%, 1.54%, 1.48%, 1.2%, 1.16%, 0.84%, and 0.78%, respectively, as shown in **Figure 3B**.

## Discussion

In this paper, we analyzed data from 32 health check centers to explore the association of lipid profiles with the prevalence of hypertension in Chinese women and the differences in lipids between hypertensive and non-hypertensive individuals. The present findings of this paper can be summarized as 1). In the overall population, TC, LDL-c, and non-HDL-c levels were higher and HDL-c levels were lower in the hypertensive individuals, while TG was not significantly different from the non-hypertensive individuals. 2). Similar results were obtained in the subgroup analyses. However, no significant difference in HDL was found between the two groups when analyzed stratified by BMI whereas no significant difference was observed between the two groups in TC, TG and LDL in the women aged >60 years. 3). Correlation analyses showed that SBP and DBP were positively correlated with TC, LDL-c, and non-HDL-c, negatively correlated with HDL-c, and not significantly correlated with TG. 4). Multivariate logistic regression analyses showed that the prevalence of hypertension increased with increasing TC, LDL-c, and non-HDL-c. In addition, the prevalence of hypertension decreased with increasing HDL-c while this association turned to be non-significant when the variables were fully adjusted. 5). BN model suggested that age, as a very important intermediate variable, indirectly affected the prevalence of hypertension. In the lipid profiles, TC and non-HDL-c indirectly affect the prevalence of hypertension as intermediate variables. Moreover, age, BMI, FPG and non-HDL-c were more important predictors for the prevalence of hypertension.

The relationship between lipid profile and hypertension may vary between populations. Choudhury et al. (23)

conducted a cross-sectional study featuring 234 Bangladeshi participants, and found that TC and TG were 1.1-fold higher, LDL 1.2-fold higher and HDL 1.1-fold lower in hypertensive patients compared to normotensives. However, their study sample size was relatively small, while the age of the participants was 38-51 years, concentrated in middle-aged adults, and information on young and old adults was missing. Recently, Yang et al. (18) showed that HDL-c was negatively associated with the prevalence of hypertension based on a cross-sectional study of totally 22,081 Chinese adults. With each 10 mg/dL increase in HDL-C, the age-adjusted blood pressure decreases by 2.4 mmHg and 1.9 mmHg for SBP and DBP, respectively. However, after adjusting for BMI, the relationship between HDL-c and hypertension prevalence shifted to a positive association. In fact, this is consistent with the findings of our previous study (14). Meanwhile, in their study, Yang et al. claimed that the negative association between HDL-c and hypertension prevalence was only observed in the young and middle-aged population, while this association was not found in the elders. In our study, 7366 elderly people were included, 5 times as many as Yang et al., and all these elderly people were apparently healthy, reducing the heterogeneity of the study population. Furthermore, Sesso et al. (8) conducted a cohort study of lipids and risk of hypertension in US women, and the results showed that elevated TC and non-HDL-c increased the risk of hypertension, whereas elevated HDL decreased the risk of hypertension. After 10.8 years of follow-up, the risk of hypertension was increased by 12% and 25% in the highest quintile of TC and Non-HDL-c groups, respectively, compared with the lowest quintile, suggesting that non-HDL-c levels are more closely related to the development of hypertension in the lipid profile, which is consistent with the results of our BN model. However, the cohort study by Tohidi et al. (24) showed that TC and non-HDL-c were not obviously associated with the development of hypertension in women from the Middle East after adjustment for maximum variables, but TG and TG/HDL-C could be useful indicators for early identification of hypertension. In addition, Akintunde et al. showed that dyslipidemia was strongly associated with the development of hypertension based on 251 Nigerian participants, with low HDL-c being the most common pattern of dyslipidemia, accounting for approximately 47.9% (25). Consistent with previous findings, dyslipidemia is strongly associated with the prevalence of hypertension, but differences in specific lipid patterns remain. In this case, it is suggested that there may be ethnic specificity in lipid profiles and the risk of hypertension in different populations. Apart from that, the sensitivity of lipid profile components to the effect of blood pressure levels varies due to genetic differences. Moreover, acquired influences such as economic level, diet, environment, and education level may play a role in reinforcing this association. In this study, we further elaborated the relationship between lipids and hypertension prevalence through subgroup analysis, multivariate logistic regression, and Bayesian modeling, validating previous studies and complementing epidemiological evidence from Asian populations. Simultaneously, the findings of this paper further emphasize the importance of routine monitoring of hypertensive patients in daily clinical practice to prevent cardiovascular diseases and other harmful consequences of hypertension.

Hypertension and dyslipidemia are important risk factors for cardiovascular disease. Approximately 80% of hypertensive patients have comorbidities such as obesity, poor glucose tolerance, and abnormal lipid metabolism (23). The mechanisms underlying dyslipidemia and the prevalence of and hypertension may exist as follows. 1). Dyslipidemia leads to endothelial dysfunction and improper vascular regulation, and nitric oxide production, release and subsequent activity are reduced in patients with high TC and low HDL-C levels (26). 2). Dyslipidemia is associated with elevated circulating levels of endothelin-1, causing the onset of hypertension. In addition, dyslipidemia could cause damage to the renal microvasculature, which is associated with the downstream effects of hypertension (27, 28). 3). Both dyslipidemia and hypertension are vital components of the metabolic syndrome and may share common pathophysiological pathways (8). 4) HDL has many anti-atherogenic properties, such as anti-inflammatory, antioxidant, anti-apoptotic and vasodilatory effects (29). 5). Insulin resistance including dyslipidemia may be a potential factor in sympathetic hyperactivity leading to hypertension, while obesity may further promote insulin resistance (30). In the multivariate logistic regression, no significant association between HDL-c and the prevalence of hypertension was observed, which seems to contradict the current understanding of HDL-c in cardiovascular diseases. Indeed, previous studies have shown that the relationship between HDL-c and hypertension may not be linear but rather curvilinear (13), which may be related to “dysfunctional HDL” (31). HDL-c levels are not equivalent to HDL-c function, which has both anti-inflammatory and pro-inflammatory properties (32). Dysfunctional HDL-c can be independently associated with HDL-c levels that increase the risk of atherosclerosis (31). Meanwhile, this shift in the relationship between HDL-c and hypertension prevalence when adjusting for BMI demonstrates that BMI may play the key role in the process of HDL-c and hypertension development, which needs to be further clarified by future studies.

To the best of our knowledge, the present study is the largest multicenter cross-sectional study exploring the relationship between lipid profile and hypertension in Chinese women. Our study is composed of young, middle-aged and older adults, reflecting the general population of the real world. In addition, the importance of patient baseline characteristics and lipid levels for the prevalence of hypertension was analyzed by BN models. The results showed that age, BMI, FPG, and non-HDL-c had a significant effect on the prevalence of hypertension, which might provide some new perspectives for future studies on hypertension.

Inevitably, this study has the following limitations. First, the design of this study is cross-sectional and reflects only the relationship between lipid profiles and the prevalence of hypertension, and no causal association can be drawn. Second, the hypertension grouping in this study was based on one office blood pressure measurement. Other than that, as information on antihypertensive medication use was missing from the patients’ baseline information, the prevalence of hypertension may be underestimated. Moreover, the category of hypertension (primary and secondary) was not differentiated. Third, the lack of information on physical activity, diet, and history of medication of chronic diseases in this study population, which remains unclear in terms of its impact on the study results. In the future, our study will be designed based on these limitations for avoiding the deficiencies. Fourth, there were significant differences in baseline information between hypertensive and non-hypertensive populations, and despite attempts to perform propensity score matching analysis for reducing the effect of confounding variables, the matching results were poor. Ultimately, a multivariate logistic regression model was selected to reduce the effect of confounding variables. However, propensity score matching had significant advantages in reducing confounders and simulating random assignment, and it is recommended that future studies should adopt this method depending on the study design and content. Fifth, the 11 cities involved in this study are economically affluent cities in China. Therefore, the present results are only representative of a portion of the Chinese population.

## Conclusion

To conclude, in Chinese women, TC, LDL-c and non-HDL-c levels were higher and HDL-c level was lower in the hypertensive population, whereas TG did not differ significantly from the non-hypertensive population. TC, LDL-c, and non-HDL-c were positively associated with prevalence of hypertension, and HDL-c was negatively associated with prevalence of hypertension but became nonsignificant after full adjustment for variables. Moreover, BN model suggested that age, BMI, FPG, and non-HDL-c had a greater effect on the development of hypertension.

## Data Availability Statement

The datasets presented in this study can be found in online repositories. The names of the repository/repositories and accession number(s) can be found in the article/supplementary material.

## Ethics Statement

Ethical review and approval was not required for the study on human participants in accordance with the local legislation and institutional requirements. Written informed consent for participation was not required for this study in accordance with the national legislation and the institutional requirements.

## Author Contributions

WKC designed this paper. WKC and GZD drafted, analyzed, interpreted this study. WKC, GZD, and YJL critically reviewed the study. All authors finally agreed to submit the manuscript.

## Funding

WKC is funded by China Scholarship Council (CSC No. 202009370095). Many thanks the CSC for its support to WKC's study and project.

## Conflict of Interest

The authors declare that the research was conducted in the absence of any commercial or financial relationships that could be construed as a potential conflict of interest.

## Publisher’s Note

All claims expressed in this article are solely those of the authors and do not necessarily represent those of their affiliated organizations, or those of the publisher, the editors and the reviewers. Any product that may be evaluated in this article, or claim that may be made by its manufacturer, is not guaranteed or endorsed by the publisher.
